# Children's health and parental socioeconomic factors: a population-based survey in Finland

**DOI:** 10.1186/1471-2458-11-457

**Published:** 2011-06-09

**Authors:** Sanna M Siponen, Riitta S Ahonen, Piia H Savolainen, Katri P Hämeen-Anttila

**Affiliations:** 1 School of Pharmacy/Social Pharmacy, University of Eastern Finland, P.O. Box 1627, FIN-70211 Kuopio, Finland; 2 Finnish Medicines Agency, Microkatu 1, Kuopio, P.O Box 55, FI-00301 Helsinki, Finland

**Keywords:** child, health, socioeconomic factors, population-based study

## Abstract

**Background:**

Socioeconomic inequalities in health are a global problem, not only among the adult population but also among children. However, studies concerning young children especially are rare. The aim of this study was to describe the health of Finnish children under 12 years of age, and the socioeconomic factors associated with health. The socioeconomic factors were parental education level, household net income, and working status.

**Methods:**

A population-based survey among Finnish children aged under 12 years (n = 6,000) was conducted in spring 2007. A questionnaire was sent to parents, and a response rate of 67% was achieved. Each child's health was explored by asking a parent to report the child's health status on a 5-point Likert scale, current symptoms from a symptoms list, and current disease(s) diagnosed by a physician. The final three outcome measures were poor health, the prevalences of psychosomatic symptoms, and long-term diseases. Data were analysed using Pearson's Chi-Square tests, and logistic regression analysis with 95% confidence intervals (CIs). P-values ≤0.05 were considered as statistically significant.

**Results:**

In total, 3% of parents reported that their child's health status was poor. The prevalences of psychosomatic symptoms and long-term diseases were both 11%. The probability for poor health status was lowest among children aged 3-6 and 7-11 years, and for psychosomatic symptoms among 3-6-year-old children, whereas the odds ratios for long-term diseases was highest among children aged 7-11 years. Parental socioeconomic factors were not associated with the children's health.

**Conclusions:**

Most of the children were reported by their parent to have good health status, and approximately one tenth had experienced some psychosomatic symptoms or long-term diseases. Our study suggests that parental socioeconomic factors are not associated with the health of children aged under 12 years in Finland.

## Background

Increasing morbidity and socioeconomic health inequalities are a global problem [1,2]. European studies have shown that low socioeconomic status, low education and low income are associated with poor health among adult populations, e.g., poor self-assessed health, mortality, prevalence of long-term diseases, and the experience of psychosomatic symptoms [3-9]. Inequalities between population groups have increased in Europe [1], including Finland [3,7], even though general well-being and living conditions have improved in recent decades [4]. Minimising health inequalities between population groups has been an objective of health policy in several European countries [1,4].

A relationship between parental socioeconomic factors and health has also been demonstrated among children. For example, parents' low education, unemployment, and low income have been associated with poor health status and an increased prevalence of chronic diseases and psychosomatic symptoms [10-19]. In addition, the presence of multiple parental socioeconomic risk factors may have a cumulative effect on children's poor health [14,18,20].

The association of low socioeconomic background and poor health is not simple. Many environmental and social factors, such as poor nutrition and poor access to health care, have been found to be associated with low socioeconomic background, e.g. low income and education, and thus with poor health and development of children [21-23]. Low education, young age and depressive symptoms of a caregiver have also been associated with poor nutrition of children, and children in such families have been found to be at developmental risk [23]. Low socioeconomic background of parents may also lead to a poor relationship with children and thereby affect their health over a longer period [22].

Some studies have reported an inverse association between parental socioeconomic status and child health. For example, in Finland the prevalence of long-term diseases among children, especially asthma and allergies, was found to be slightly higher in families with a highly educated mother than in families with a less educated mother [24]. In contrast, family income was not associated with long-term diseases among children. An increased number of diseases diagnosed by a physician and colds among children with highly educated parents have been reported in West Germany [25], and in the United States for respiratory conditions [16]. In addition, a similar relationship between parental occupation and psychosomatic symptoms has been found in Scotland, especially among girls [26].

Age dependency in health inequalities have also been a point of interest for several researchers [16,17,26]. On the basis of equalization theory it has been hypothesized that health inequalities are more evident in childhood, and diminish in adolescence [26]. However, this is not supported by the results of other studies [10,13,16,17,26]. In fact, it remains unclear whether parental socioeconomic background is associated with children's health at different ages.

Clarifying the association of parental socioeconomic background and children's health is important since it has been shown that health inequalities in childhood predict poor health in adulthood [27,28]. Most of the studies have focused on health as self-reported by the parent [12,14,17], the prevalence of long-term diseases [24], physical and psychosomatic symptoms [27], or combinations of these [10,11,13,15,16]. Studies have focused mainly on children under 18 years of age as one homogenous group [11,14,16,18-20], and a few on children over 10 years of age [26,29]. Only a few studies have focused on children aged under 18 years by using different age groups [10,13,17]. To our knowledge, studies using three dimensions (self-reported health, prevalence of long-term diseases, and psychosomatic symptoms) are rare [26], especially among young children.

The aim of this study was to describe the health of Finnish children aged under 12 years, and socioeconomic factors associated with health. The socioeconomic factors were parental education level, household net income, and working status.

## Methods

### Context

#### Socioeconomic characteristics in Finland

In 2007, when this study was conducted, there were about 5.3 million inhabitants in Finland, of whom 17% (n = 894,590) were children under 15 years of age (Statistics Finland, http://www.stat.fi/). Seventy percent of 15-64-year-old people were working and 6% were unemployed. In addition, 65% of those aged over 14 years had some educational qualification obtained after primary school, mostly in senior high school or vocational school (11-13 years of education) (39%). Polytechnic, college or university degrees (≥ 15 years of education) had been completed by 26% of the population. Furthermore, 14% of the total population were in the low-income group, living below 60% of the median income level (€13,600/year/one-person-household in 2007). Being in the low-income group was most common among people who were unemployed, students, or retired.

#### Health care in Finland

In Finland, primary health care is available for all citizens for a small fee. In addition, special health care is offered in 20 hospital districts. The primary and special health care systems are complemented by private health care, and a part of the fees are reimbursed by the Social Insurance Institution.

The Finnish government provides up to 9 months paid maternity leave [30]. Partial support from the government is also provided for up to three years, if a parent wishes to take care of a child at home. Children's health is monitored regularly, free of charge, in child welfare clinics and in the school health care system until the end of primary school (Decision of the Council of State 380/2009). More information about the health care system in Finland may be found on the websites of the Social Insurance Institution (http://www.kela.fi) and the Ministry of Social Affairs and Health (http://www.stm.fi).

### Data collection

We carried out a population-based survey among Finnish children aged under 12 years in spring (February-April) 2007. The study sample consisted of 6,000 Finnish children living in Finland in December 2006, and they were randomly selected from the Finnish National Population Register Centre, excluding children in institutional care.

Data were collected using a structured questionnaire consisting of 30 questions about background information, health status, and the use of medicine by the child (Additional File 1 Additional File 2). Furthermore, questions regarding information sources about medicines and background information about the respondent were included. The questions about children's health were designed to be consistent with questions used in previous population-based studies conducted in Finland [3,31], and with the school health surveys among children aged 14-18 years which are conducted biennially [32]. The questionnaire was available in Finnish and Swedish, both of which are official languages in Finland, and it was first pilot-tested for face-validity among a convenience sample of 61 mothers. Minor modifications were made based on the pilot.

The questionnaire was sent to parents, primarily to mothers, but it was addressed to the parent who usually takes care of the child's medication. The child's name was printed on the questionnaire to indicate the child that was selected from the study population. This was important in families with more than one child. Two reminders were sent. After the questionnaires were returned, the child's name was removed and a random number was assigned to each questionnaire to ensure the anonymity of the respondent. This was ensured by following national and local instructions for researchers (National Advisory Board on Ethics: http://www.tenk.fi/ENG/function.htm). A more detailed description of the study sample and data collection is presented elsewhere [33].

### Outcomes

The main outcomes of this study were poor health among children, and the prevalence of psychosomatic symptoms and long-term diseases.

Health status was determined by asking a parent to report the current health status of their child using the following categories: good, fairly good, moderate, fairly poor and poor. For the purpose of the data analysis, categories were combined as "moderate or poor" (including moderate n = 106, fairly poor n = 25 and poor n = 4), and as "good" (including fairly good n = 631 and good n = 3,249).

The prevalence of psychosomatic symptoms was measured via a list of symptoms (described in Table 1). Parents were asked to circle "yes" or "no" whether their child currently experienced that symptom. They could also select "don't know" or report other symptoms if they were not in the list. In the symptoms list, sleep disorders, fatigue or dizziness, anxiety and depression were considered to be psychosomatic symptoms. Most of the children who had experienced psychosomatic symptoms had experienced one psychosomatic symptom (8% of children), and only 3% of children had experienced two psychosomatic symptoms or more. Thus, the final outcome included children who had experienced one or more psychosomatic symptoms.

**Table 1:** Current symptoms experienced by children as reported by the parent

Symptoms	Boys			Girls			Total
	**0-2**	**3-6**	**7-11**	**0-2**	**3-6**	**7-11**	
	**(n = 532)**	**(n = 677)**	**(n = 865)**	**(n = 468)**	**(n = 595)**	**(n = 847)**	**(n = 3984)**
	**n (%)**	**n (%)**	**n (%)**	**n (%)**	**n (%)**	**n (%)**	**n (%)**

**Psychosomatic symptoms**	66 (12.4)	50 (7.4)	104 (12.0)	67 (14.3)	47 (7.9)	103 (12.2)	437 (10.9)
Sleep disorders	50 (9.4)	26 (3.8)	29 (3.4)	54 (11.5)	23 (3.9)	26 (3.1)	218 (5.5)
Fatigue or dizziness	24 (4.5)	21 (3.1)	50 (5.8)	21 (4.9)	19 (3.2)	61 (7.2)	196 (4.9)
Anxiety	3 (0.6)	12 (1.8)	43 (5.0)	7 (1.5)	12 (2.0)	37 (4.4)	114 (2.9)
Depression	0 (0)	3 (0.4)	16 (1.8)	1 (0.2)	4 (0.7)	11 (1.3)	35 (0.9)
							
**Other symptoms**	382 (71.8)	421 (62.2)	507 (58.6)	318 (67.9)	386 (64.9)	551 (65.1)	2565 (64.4)
Symptoms of common cold	244 (45.9)	255 (37.7)	222 (25.7)	190 (40.6)	209 (35.1)	224 (26.4)	1344 (33.7)
Eczema	156 (29.3)	143 (21.1)	163 (18.8)	135 (28.8)	170 (28.6)	181 (21.4)	948 (23.8)
Flatulence	67 (12.6)	54 (7.8)	110 (12.7)	48 (10.3)	47 (7.9)	128 (15.1)	454 (11.4)
Growing pains	11 (2.1)	78 (11.5)	98 (11.3)	4 (0.9)	57 (9.6)	108 (12.8)	356 (8.9)
Sore throat	20 (3.8)	25 (3.7)	55 (6.4)	7 (1.5)	38 (6.4)	72 (8.5)	217 (5.4)
Headache	1 (0.2)	19 (2.8)	71 (8.2)	1 (0.2)	12 (2.0)	102 (12.0)	206 (5.2)
Fever	32 (6.0)	31 (4.6)	23 (2.6)	31 (6.6)	25 (4.2)	13 (1.5)	155 (3.9)
Allergy symptoms	15 (2.8)	19 (2.8)	42 (4.9)	15 (3.2)	15 (2.5)	38 (4.5)	144 (3.6)
Constipation	24 (4.5)	23 (3.4)	16 (1.8)	25 (5.3)	21 (3.5)	25 (3.0)	134 (3.4)
Earache	37 (7.0)	21 (3.1)	12 (1.4)	20 (4.3)	21 (3.5)	17 (2.0)	128 (3.2)
Pain in neck or shoulders	0 (0)	5 (0.7)	26 (3.0)	0 (0)	2 (0.3)	50 (5.9)	83 (2.1)
Diarrhoea	16 (3.0)	12 (1.8)	4 (0.5)	14 (3.0)	6 (1.0)	7 (0.8)	59 (1.5)
Stomach flu	9 (1.7)	11 (1.6)	5 (0.6)	9 (1.9)	4 (0.7)	6 (0.7)	44 (1.1)
Low back pain	0 (0)	6 (0.9)	10 (1.2)	0 (0)	0 (0)	16 (1.9)	32 (0.8)
Other symptoms*	42 (7.9)	46 (6.8)	72 (8.3)	31 (6.6)	49 (8.2)	88 (10.4)	328 (8.2)

*e.g. other stomach symptoms and other symptoms of pain

The prevalence of long-term diseases was explored by asking the parent to write down any diseases or injuries which had been diagnosed by a physician that their child currently had. Of those diseases reported by parents (shown in Table 2), asthma, allergy, epilepsy, diabetes, lactose intolerance, migraine, attention deficit hyperactivity disorder (ADHD), heart defect, atopy, rheumatoid arthritis, and celiac disease were considered long-term diseases. The majority of children with a long-term disease had one long-term disease (9% of children), and 2% of children had two or more long-term diseases. Therefore, the final outcome included children who had one or more long-term diseases.

**Table 2:** Children's diseases diagnosed by a physician, as reported by the parent

Diseases	Boys			Girls			Total
	**0-2**	**3-6**	**7-11**	**0-2**	**3-6**	**7-11**	
	**(n = 532)** **n (%)**	**(n = 680)** **n (%)**	**(n = 879)** **n (%)**	**(n = 467)** **n (%)**	**(n = 599)** **n (%)**	**(n = 852)** **n (%)**	**(n = 4009)** **n (%)**

**Long-term diseases**	53 (10.0)	76 (11.2)	127 (14.4)	40 (8.6)	55 (9.2)	105 (12.3)	456 (11.4)
Allergy	15 (2.8)	26 (3.8)	49 (5.6)	16 (3.4)	16 (2.7)	44 (5.2)	166 (4.1)
Asthma	16 (3.0)	36 (5.3)	42 (4.8)	7 (1.5)	16 (2.7)	35 (4.1)	152 (3.8)
Atopy	22 (4.1)	19 (2.8)	20 (2.3)	17 (3.6)	25 (4.2)	14 (1.6)	117 (2.9)
Migraine	0 (0)	0 (0)	12 (1.4)	0 (0)	3 (0.5)	9 (1.1)	24 (0.6)
ADHD	0 (0)	3 (0.4)	17 (1.9)	0 (0)	0 (0)	2 (0.2)	22 (0.5)
Diabetes	0 (0)	2 (0.3)	4 (0.5)	2 (0.4)	0 (0)	9 (1.1)	17 (0.4)
Heart defect	5 (0.9)	3 (0.4)	5 (0.6)	3 (0.6)	1 (0.2)	3 (0.4)	20 (0.5)
Epilepsy	1 (0.2)	3 (0.4)	2 (0.2)	1 (0.2)	3 (0.5)	2 (0.2)	12 (0.3)
Lactose intolerance	0 (0)	2 (0.3)	2 (0.2)	0 (0)	2 (0.3)	3 (0.4)	9 (0.2)
Rheumatoid arthritis	0 (0)	1 (0.2)	0 (0)	0 (0)	0 (0)	5 (0.6)	6 (0.1)
Celiac disease	1 (0.2)	0 (0.2)	2 (0.2)	0 (0)	1 (0.2)	1 (0.1)	5 (0.1)
							
**Impairments**	5 (0.9)	19 (2.8)	34 (3.9)	2 (0.4)	4 (0.7)	24 (2.8)	88 (2.2)
Visual impairment/heterotropia	2 (0.4)	6 (0.9)	3 (0.3)	1 (0.2)	3 (0.5)	7 (0.8)	22 (0.5)
Dysphasia	0 (0)	7 (1.0)	11 (1.3)	0 (0)	0 (0)	4 (0.5)	22 (0.5)
Mentally disabled	2 (0.4)	3 (0.4)	7 (0.8)	1 (0.2)	0 (0)	3 (0.4)	16 (0.4)
Hearing impairment	0 (0)	0 (0)	3 (0.3)	0 (0)	0 (0)	1 (0.1)	4 (< 0.1)
Other impairment*	1 (0.2)	5 (0.7)	14 (1.6)	0 (0)	1 (0.2)	10 (1.2)	31 (0.8)
							
**Other diseases**	60 (11.3)	36 (5.3)	49 (5.6)	27 (5.8)	33 (5.5)	41 (4.8)	246 (6.1)
Otitis	28 (5.3)	10 (1.5)	4 (0.5)	14 (3.0)	10 (1.2)	3 (0.4)	69 (1.7)
Other infection‡	10 (1.9)	6(0.9)	9 (1.0)	5 (1.1)	5 (0.8)	6 (0.7)	41 (1.0)
Urticaria/other skin rash	1 (0.2)	2 (0.3)	4 (0.5)	1 (0.2)	2 (0.3)	4 (0.5)	14 (0.3)
Common cold	5 (0.9)	0 (0)	0 (0)	1 (0.2)	3 (0.5)	1 (0.1)	10 (0.2)
Other†	26 (4.9)	20 (2.9)	34 (3.9)	10 (2.1)	15 (2.5)	28 (3.3)	118 (2.9)

*including autism ‡including other respiratory tract infections †including other diseases diagnosed by a physician, e.g., varicella

Parental socioeconomic factors were measured by asking the parent who completed the questionnaire to report their highest educational level from a 7-item list, and household net income per month (€) from an 11-items list (scale: under €500 to over €10,000). For the analysis, the highest educational level and household net income per month were categorized as shown in Table 3. Working status of a parent was classified as "Working" and "Not in work" (including unemployed parents, home with children, retired, studying, or on sick leave), which also made it possible to avoid multicollinearity with the household net income.

**Table 3:** Characteristics of the study population (n = 4,032)

	Characteristics of the study population	Health status			Prevalence of psychosomatic symptom(s)		Prevalence of long-term disease(s)	
		Good	Moderate or poor					
	**n (%)**	**n (%)**	**n (%)**	p*	**n (%)**	p*	**n (%)**	p*

**Gender**				0.81		0.45		0.07
Boy	2106 (52)	2023 (97)	69 (3)		220 (11)		256 (12)	
Girl	1926 (48)	1857 (97)	66 (3)		217 (11)		200 (10)	
**Age**				0.00		0.00		0.00
0-2	1004 (25)	932 (94)	65 (7)		133 (13)		93 (9)	
3-6	1287 (32)	1256 (98)	28 (2)		97 (8)		131 (10)	
7-11	1741 (43)	1692 (98)	42 (2)		207 (12)		232 (13)	
**Parent's highest level of education**				0.80		0.89		0.21
Junior high school or less (≤9 years)	252 (6)	244 (97)	7 (3)		29 (12)		23 (9)	
Senior high school/vocational school (11-13 years)	2456 (61)	2365 (97)	85 (4)		265 (11)		269 (11)	
Polytechnic, college or university degree (≥ 15 years)	1291 (32)	1240 (97)	41 (3)		137 (11)		161 (13)	
**Household's net income/month (€)**				0.55		0.05		0.60
Below 1999	797 (20)	766 (96)	30 (4)		101 (13)		93 (12)	
2000-2999	1292 (32)	1243 (97)	45 (4)		151 (12)		134 (10)	
3000-3999	1419 (35)	1372 (97)	39 (3)		131 (9)		170 (12)	
4000-10,000	347 (9)	336 (97)	10 (3)		35 (10)		38 (11)	
**Working status**				0.04		0.01		0.06
Working	2541 (63)	2460 (97)	73 (3)		248 (10)		305 (12)	
Not in work	1470 (37)	1402 (96)	60 (4)		185 (13)		148 (10)	

* p-value of ≤0.05 considered statistically significant

### Statistical analysis

Data were analyzed with SPSS for Windows, Version 14.0 (SPSS Inc., Chigaco, IL, USA). The results are presented using descriptive statistics: frequencies, percentages and cross-tabulation. Pearson's Chi-Square test was used when exploring the association between categorical variables. A p-value of ≤0.05 was considered to be statistically significant. Three age groups of children (0-2, 3-6 and 7-11 years) were used in analyses, as in previous population-based studies conducted in Finland [31]. Logistic regression analyses were conducted when measuring the association between parental socioeconomic background and the child's health. The results are presented as odds ratios (ORs) together with their 95% confidence intervals (CIs).

The discriminating ability of the logistic regression model was measured with ROC analysis, which showed the discriminating ability of the model to be excellent for poor health (AUROC value 0.901), moderate for psychosomatic symptoms (0.742), and adequate for long-term diseases (0.675). The interaction between age and gender was also measured but no significant results were found. Thus, the interaction variable of age and gender was not included in the analysis.

## Results

The final study population consisted of 5,992 children, because the parents of 8 children were not reached by mail. We received 4,121 completed questionnaires. Eighty-nine questionnaires were excluded because they were completed for another child than the child included in the study sample. Thus, the final study sample was 4,032 (response rate 67%). The respondent was mainly the mother (in 95% of cases) and secondly the father (in 4% of cases). We analysed the representativeness of the study population and non-respondents, which showed that the study population was representative of the target population in respect of age and gender. A more specific analysis of the representativeness of the study population is available elsewhere [34].

The characteristics of the study population are shown in Table 3. Most parents reported that their child had good health, while moderate or poor health status was reported for 3% of the children. The prevalences of psychosomatic symptoms (11%) and long-term diseases (11%) were equal. Of the socioeconomic factors, most of the parents (61%) had senior high school or vocational school (11-13 years) as their highest educational background, and the majority of the parents were working (63% of parents). Most parents had €2,000-2,999 (32% of parents) or €3,000-3,999 (35%) as their household net income per month.

Tables 1 and 2 show the symptoms children had experienced, and the diseases diagnosed by a physician, according to age and gender. Symptoms of the common cold and eczema were the most prevalent symptoms among the children (34% and 24% of children, respectively), and especially among children aged 0-2 years (Table 1). Boys were more likely to have experienced symptoms of the common cold at this age than girls (46% of boys and 41% of girls), but the symptoms of eczema were equally common among both genders. Sleep disorders were the most commonly reported psychosomatic symptoms (6% of children), and they were most prevalent among children aged 0-2 years (9% of boys and 12% of girls), decreasing with age. In contrast, the most common diseases diagnosed by a physician were allergy (4% of children) and asthma (4%) (Table 2). These were also the most common long-term diseases. The prevalence of these long-term diseases increased with age, and they were nearly equally common among boys and girls at 7-11 years of age.

Of the factors measured, age was found to be associated with poor health status, prevalence of psychosomatic symptoms, and long-term diseases (Table 3). Of the socioeconomic factors, parents' education level or household net income per month were not associated with any of the health variables of the child, whereas working status was associated with poor health status and the prevalence of psychosomatic symptoms. Poor health status was most commonly reported for children aged 0-2 years (7%), and psychosomatic symptoms for children aged 0-2 (13%) and 7-11 years (12%). In contrast, the prevalence of long-term diseases increased with age, being most common among children aged 7-11 years (13%). Poor health status and psychosomatic symptoms were more common among children whose parent was not working (4% for poor health status, and 13% for psychosomatic symptoms) compared with those whose parent was working (3% and 10%, respectively).

Only age was significantly associated with poor health, psychosomatic symptoms and the prevalence of long-term diseases in the multivariate analysis when adjusted for other variables (Table 4). The ORs for poor health were lower among children aged 3-6 (OR 0.35, CI 0.20-0.61) and 7-11 years (OR 0.33, CI 0.20-0.57), and the ORs for psychosomatic symptoms were lower among 3-6-year-old children (OR 0.53, CI 0.39-0.72) compared with children aged 0-2 years. In contrast, the ORs for long-term diseases were highest among 7-11-year-old children (OR 1.67, CI 1.24-2.23).

**Table 4:** Logistic regression model of poor health status among children (n = 4032), psychosomatic symptoms and long-term diseases†

Variable	Moderate or poor health status	Psychosomatic symptoms	Long-term diseases
	OR (Cl 95%)	OR (Cl 95%)	OR (Cl 95%)
**Child**			
Gender			
Girl	1.00	1.00	1.00
Boy	0.90 (0.59-1.37)	0.92 (0.74-1.14)	1.23 (1.00-1.514)
Age, years			
0-2	1.00	1.00	1.00
3-6	0.35 (0.20-0.61)	0.53 (0.39-0.72)	1.22 (0.90-1.66)
7-11	0.33 (0.20-0.57)	0.93 (0.71-1.23)	1.67 (1.24-2.23)
**Parent**			
Parent's highest level of education			
Polytechnic, college or university degree (≥ 15 years)	1.00	1.00	1.00
Senior high school/vocational school (11-13 years)	1.23 (0.76-1.98)	1.07 (0.83-1.37)	0.78 (0.62-0.98)
Junior high school or less (≤9 years)	0.89 (0.32-2.50)	1.16 (0.72-1.85)	0.63 (0.38-1.04)
			
Household's net income/month (€)			
4000-10, 000	1.00	1.00	1.00
3000-3999	0.88 (0.38-2.06)	0.78 (0.52-1.19)	1.26 (0.85-1.86)
2000-2999	1.32 (0.57-3.10)	0.97(0.63-1.48)	1.12 (0.74-1.69)
Below 1999	1.42 (0.58-3.49)	0.98 (0.62-1.54)	1.25 (0.81-1.94)
Working status			
Working	1.00	1.00	1.00
Not in work	0.77 (0.48-1.22)	1.22 (0.96-1.56)	0.88 (0.69-1.12)

†Adjusted for number of long-term diseases, prevalence of impairments, prevalence of other diseases, the number of psychosomatic symptoms, and the number of other symptoms

## Discussion

The majority of the children were reported by their parent to have good health. Approximately one tenth had experienced a psychosomatic symptom, or had a long-term disease, most commonly allergies or asthma. Allergies and asthma were also the most common long-term diseases among children reported in earlier studies conducted in Finland [3,20], and are among the most frequently reported long-term diseases also internationally [13,17,19]. In contrast to most previous studies, we found that parental socioeconomic factors were not associated with the health of Finnish children aged under 12 years.

Some of the findings concerning the association of children's health and socioeconomic factors which contradict those reported in the literature may be due to the different methodologies used in the studies. One of the reasons for these differences may be that most of the studies included children under 18 years of age as one homogenous group [11,14,16,18-20], and thus it is difficult to estimate whether there are differences in the association with parental socioeconomic background and children's health at different ages. Our results may suggest that parental socioeconomic status is a more important determinant of health in adolescence than in childhood, and that in childhood the effect of parental socioeconomic background on children's health may still be prevented. Prenatal care, child welfare clinics, and school health care where children's health is monitored regularly in Finland, are likely to have a positive effect on their health. This hypothesis is supported by the findings from a previous study in Finland, which found only a minor increase in the prevalence of long-term diseases among children with highly educated mothers [20]. The impact of health care on children's health was, however, not the focus of our study, which remains an important topic for further studies.

The relationship between socioeconomic factors and children's health has been widely studied. However, the social environment, family atmosphere, and relationships with parents and friends may also have an important impact on the health of children [19,29]. For example, poor relationships with parents and friends and poor parental mental health have been found to predict poor health of children. Experiencing warmth and control from mothers has been found to improve children's health [14]. Furthermore, this has been found to be closely connected with parental socioeconomic status; mothers with high income who are highly educated have been shown to have greater abilities to control and give warmth to a child than mothers with low income or less education. Furthermore, one study showed that the relationship between family poverty and depressive symptoms among children were mostly explained by the family environment (poor parental support, divorce or separation) [35]. The parents in our study were mostly well educated and had a good income, and the majority were working, which may explain partly why we did not find an association between parental socioeconomic background and the health of the children.

Our study describes the health of a representative sample of Finnish children aged less than 12 years from three dimensions: self-rated health by the parent, the prevalence of long-term diseases, and psychosomatic symptoms. It was not possible to determine the parental socioeconomic factors among the non-respondents, which is one of the limitations of our study. In addition, there are some distinctions in the definitions of long-term diseases and psychosomatic symptoms between previous studies and our study, which may affect the comparison of the results. For example, we were not able to estimate the severity of long-term diseases or psychosomatic symptoms, as has been done in two previous studies in Nordic countries [10,11]. Our study focused on the association with parental socioeconomic background and children's health. Further studies are needed to explore how different environmental factors, such as family structure, family relations and nutrition, are associated with socioeconomic background and children's health in Finland.

## Conclusions

Most of the Finnish children under 12 years of age were reported by their parent to have good health, and approximately one tenth had experienced psychosomatic symptoms or long-term diseases. In contrast to most previous studies, our study found that parental socioeconomic status is not associated with the health of Finnish children aged less than 12 years. The results may indicate that at this age, the effect of low parental socioeconomic background on children's health may still be prevented.

## Competing interests

The authors declare that they have no competing interests.

## Authors' contributions

SS drafted the manuscript and participated in the acquisition, analysis and interpretation of the data. RA and KH-A have made substantial contributions to the conception, design and acquisition the data, and designing and revising the manuscript. PS has been involved in designing and revising the manuscript. All authors have read and approved the final manuscript.

## Pre-publication history

The pre-publication history for this paper can be accessed here:

http://www.biomedcentral.com/1471-2458/11/457/prepub

## Supplementary Material

Additional file 1 **Survey questionnaire**. Original questionnaire used in the study in Finnish Click here for file

Additional file 2 Survey questionnaire Original questionnaire used in the study, translated into English Click here for file
