# Supplementary material for: Children's health and parental socioeconomic factors: a population-based survey in Finland
Source: BMC Public Health. 2011 Jun 9;11:457. doi: 10.1186/1471-2458-11-457 (PMC3135536; doi:10.1186/1471-2458-11-457)
Supplement: Additional file 1 — Survey questionnaire. Original questionnaire used in the study in Finnish [file 1471-2458-11-457-S1.DOC]

Kuopion yliopisto

Sosiaalifarmasian laitos

Maaliskuu 2007

**LAPSEN LÄÄKKEIDEN KÄYTTÖ**

**Vastaa kysymyksiin rengastamalla sopivat vastausvaihtoehdot tai kirjoittamalla vastauksesi sitä varten varattuun tilaan. Vastauksesi antaa arvokasta tietoa, vaikka lapsella ei olisikaan käytössään mitään lääkettä.**

**LAPSEN TAUSTATIEDOT**

**1. Onko lapsi**

1. Tyttö
2. Poika

**3. Onko lapsi**

1. Esikoinen
2. Toinen lapsi
3. Kolmas lapsi
4. Muu. Kuinka mones? _____________________________

**2. Lapsen syntymäaika**

päivä kuukausi vuosi

**4. Miten lapsen päivähoito on järjestetty, vai käykö hän koulua?**

1. Käy koulua tai esikoulua
2. Perhepäivähoidossa tai kolmiperhehoidossa
3. Päiväkodissa
4. Hoidetaan kotona yksin, sisarusten tai hoitolasten kanssa
5. Muulla tavalla. Miten? ____________________________

______________________________________________

**LAPSEN TERVEYDENTILA**

**5. Millainen on mielestäsi lapsen tämänhetkinen terveydentila?**

1. Hyvä
2. Melko hyvä
3. Keskitasoinen
4. Melko huono
5. Huono

**6. Onko lapsella tällä hetkellä jokin lääkärin toteama sairaus tai vamma?**

1. Ei
2. Kyllä. Mikä / mitkä? ______________________________

**______________________________________________**

**7. Alla on lueteltu joukko oireita. Merkitse jokaisen oireen kohdalle, onko lapsella niitä tällä hetkellä.**

| **Oireet** | **Ei** | **Kyllä** | | **En osaa**  **sanoa** |
| --- | --- | --- | --- | --- |
|  |  |  | |  |
| Ummetus | 1 | 2 | | 3 |
| Ripuli | 1 | 2 | | 3 |
| Oksennustauti | 1 | 2 | | 3 |
| Ilmavaivat | 1 | 2 | | 3 |
| Muut vatsaoireet, mitkä? __________________________ | 1 | 2 | | 3 |
| Päänsärky | 1 | 2 | | 3 |
| Niska- tai hartiakipu | 1 | 2 | | 3 |
| Selän alaosan kipu | 1 | 2 | | 3 |
| Korvakipu tai korvatulehdus | 1 | 2 | | 3 |
| Kurkkukipu | 1 | 2 | | 3 |
| Kasvukipu | 1 | 2 | | 3 |
| Muu kipu, mikä? ________________________________ | 1 | 2 | | 3 |
| Kuume | 1 | 2 | | 3 |
| Flunssan oireet (esim. nuha, yskä) | 1 | 2 | | 3 |
| Allergiaoireet (esim. nuha, silmäoireet) | 1 | 2 | | 3 |
| Ihottuma tai iho-oireet | 1 | 2 | | 3 |
| Väsymys tai heikotus | 1 | 2 | | 3 |
| Univaikeudet | 1 | 2 | | 3 |
| Jännittyneisyys tai hermostuneisuus | 1 | 2 | | 3 |
| Alakuloisuus tai masentuneisuus | 1 | 2 | | 3 |
| Muu oire, mikä? ________________________________ | 1 | 2 | | 3 |
|  |  | |  | |

**LAPSEN LÄÄKKEIDEN KÄYTTÖ**

**8. Onko lapsella tällä hetkellä käytössä mitään lääkärin määrää-mää lääkettä?**

1. Ei (siirry kysymykseen numero 9)
2. Kyllä. Kirjaa kaikki käytössä olevat lääkkeet ja niiden käyttötarkoitus, myös tarvittaessa otettavat lääkkeet. Voit tarvittaessa jatkaa kyselylomakkeen viimeiselle sivulle.

| Lääkkeen nimi  (esim. Beclomet Easyhaler) |  | Käyttötarkoitus  (esim. astma) |
| --- | --- | --- |
|  |  |  |
|  |  |  |
|  |  |  |
|  |  |  |
|  |  |  |
|  |  |  |
|  |  |  |

**10. Käyttääkö lapsi jotain ilman reseptiä saatavaa lääkettä tai vitamiinia päivittäin tai lähes päivittäin?**

1. Ei (siirry kysymykseen numero 11)
2. Kyllä. Kirjaa kaikki käytössä olevat lääkkeet ja/tai vitamiinit ja kuinka kauan käyttö on jatkunut.

| Valmisteen nimi |  | Kuinka kauan käyttänyt? |
| --- | --- | --- |
|  |  |  |
|  |  |  |
|  |  |  |
|  |  |  |
|  |  |  |

**12. Onko jokin lääke joskus aiheuttanut lapselle haittaa?**

1. Ei (siirry kysymykseen numero 13)
2. Kyllä. Kirjaa kaikki haitat. Luettele, mitkä lääkkeet ovat niitä aiheuttaneet. Voit tarvittaessa jatkaa kyselylomakkeen viimeiselle sivulle.

________________________________________________________

________________________________________________________

________________________________________________________

________________________________________________________

________________________________________________________

**9. Onko lapsi käyttänyt eilen tai toissapäivänä mitään apteekis-ta ilman reseptiä saatavaa lääkettä, mukaan lukien vitamiinit?**

1. Ei (siirry kysymykseen numero 10)
2. Kyllä. Kirjaa kaikki käytössä olevat lääkkeet ja/tai vitamiinit ja niiden käyttötarkoitus.

| Lääkkeen nimi  (esim. Pantyson) |  | Käyttötarkoitus  (esim. ihottuma) |
| --- | --- | --- |
|  |  |  |
|  |  |  |
|  |  |  |
|  |  |  |
|  |  |  |
|  |  |  |
|  |  |  |

**11. Onko lapsi käyttänyt eilen tai toissapäivänä ilman reseptiä saatavaa luontaistuotetta, rohdosvalmistetta ja/tai homeo-paattista valmistetta?**

1. Ei (siirry kysymykseen numero 12)
2. Kyllä. Kirjaa kaikki käytössä olevat valmisteet ja niiden käyttötarkoitus.

| Valmisteen nimi |  | Käyttötarkoitus |
| --- | --- | --- |
|  |  |  |
|  |  |  |
|  |  |  |
|  |  |  |
|  |  |  |

**13. Onko lapsen lääkehoidossa joskus ollut muita ongelmia?**

1. Ei (siirry kysymykseen numero 14)
2. Kyllä. Kirjaa kaikki ongelmat. Luettele, mitkä lääkkeet ovat niitä aiheuttaneet. Voit tarvittaessa jatkaa kyselylomak-keen viimeiselle sivulle.

______________________________________________________

______________________________________________________

______________________________________________________

______________________________________________________

______________________________________________________

**14. Minkä ikäisenä lapsi voi mielestäsi itsenäisesti ilman vanhemman neuvoja ja valvontaa päättää lääkkeen käytöstä pieneen vaivaan, esimerkiksi päänsärkyyn tai muuhun vastaavaan?**

1 Alle 4-vuotiaana 4 8–9-vuotiaana 7 14–15-vuotiaana

2 4–5-vuotiaana 5 10–11-vuotiaana 8 16–17-vuotiaana

3 6–7-vuotiaana 6 12–13-vuotiaana 9 18-vuotiaana tai vanhempana

10 En osaa sanoa

**TIETOLÄHTEIDEN KÄYTTÖ**

**15. Alla on lueteltu joukko tietolähteitä. Ympyröi jokaisen tietolähteen kohdalle, kuinka paljon olet käyttänyt kyseistä lähdettä lapsen lääkitykseen liittyvissä asioissa.**

| **Tietolähde** | **Paljon** | **Jonkin verran** | **Vähän** | **En lainkaan** |
| --- | --- | --- | --- | --- |
|  |  |  |  |  |
| Lääkäri | 3 | 2 | 1 | 0 |
| Sairaanhoitaja | 3 | 2 | 1 | 0 |
| Terveydenhoitaja (neuvolassa, koulussa) | 3 | 2 | 1 | 0 |
| Sukulainen tai ystävä, jolla on terveydenhuoltoalan koulutus | 3 | 2 | 1 | 0 |
| Apteekin henkilökunta | 3 | 2 | 1 | 0 |
| Puhelinpalvelu, mikä? ______________________________________ | 3 | 2 | 1 | 0 |
| Lääkepakkauksen sisällä oleva pakkausseloste | 3 | 2 | 1 | 0 |
| Lääkkeisiin liittyvät esitteet | 3 | 2 | 1 | 0 |
| Lääkärikirja | 3 | 2 | 1 | 0 |
| Muu kirja, mikä? ___________________________________________ | 3 | 2 | 1 | 0 |
| Terveysalan lehdet | 3 | 2 | 1 | 0 |
| Tavalliset sanoma- ja aikakausilehdet | 3 | 2 | 1 | 0 |
| Radio, TV | 3 | 2 | 1 | 0 |
| Internet, mikä/mitkä sivu(t)?__________________________________ | 3 | 2 | 1 | 0 |
| Sukulaiset, ystävät ja tuttavat | 3 | 2 | 1 | 0 |
| Luontaistuotekauppa | 3 | 2 | 1 | 0 |
| Muu, mikä? _______________________________________________ | 3 | 2 | 1 | 0 |
|  | | | | |

**16. Miten luotettavina pidät seuraavia tietolähteitä lapsen lääkitykseen liittyvissä asioissa?**

| **Tietolähde** | **Erittäin luotettava** | **Luotettava** | | | **Melko**  **luotettava** | **Ei**  **luotettava** | | | **En osaa sanoa tai en ole käyttänyt** |
| --- | --- | --- | --- | --- | --- | --- | --- | --- | --- |
|  |  |  | | |  |  | | |  |
| Lääkäri | 4 | 3 | | | 2 | 1 | | | 0 |
| Sairaanhoitaja | 4 | 3 | | | 2 | 1 | | | 0 |
| Terveydenhoitaja (neuvolassa, koulussa) | 4 | 3 | | | 2 | 1 | | | 0 |
| Sukulainen tai ystävä, jolla on terveydenhuoltoalan koulutus | 4 | 3 | | | 2 | 1 | | | 0 |
| Apteekin henkilökunta | 4 | 3 | | | 2 | 1 | | | 0 |
| Puhelinpalvelu, mikä? ______________________________ | 4 | 3 | | | 2 | 1 | | | 0 |
| Lääkepakkauksen sisällä oleva pakkausseloste | 4 | 3 | | | 2 | 1 | | | 0 |
| Lääkkeisiin liittyvät esitteet | 4 | 3 | | | 2 | 1 | | | 0 |
| Lääkärikirja | 4 | 3 | | | 2 | 1 | | | 0 |
| Muu kirja, mikä? __________________________________ | 4 | 3 | | | 2 | 1 | | | 0 |
| Terveysalan lehdet | 4 | 3 | | | 2 | 1 | | | 0 |
| Tavalliset sanoma- ja aikakausilehdet | 4 | 3 | | | 2 | 1 | | | 0 |
| Radio, TV | 4 | 3 | | | 2 | 1 | | | 0 |
| Internet, mikä sivu?________________________________ | 4 | 3 | | | 2 | 1 | | | 0 |
| Sukulaiset, ystävät ja tuttavat | 4 | 3 | | | 2 | 1 | | | 0 |
| Luontaistuotekauppa | 4 | 3 | | | 2 | 1 | | | 0 |
| Muu, mikä? ______________________________________ | 4 | 3 | | | 2 | 1 | | | 0 |
|  |  | |  |  | | |  |  | |

**TAUSTATIEDOT**

**17. Tämän lomakkeen kysymyksiin vastasi**

1. Äiti
2. Isä
3. Joku muu. Kuka? __________________________­­­­_______

**19. Koko perheen lasten lukumäärä** ________________________

**21. Osallistuiko lapsi kyselylomakkeen täyttämiseen?**

1. Ei
2. Kyllä

**23. Missä läänissä asut?**

1. Lapin läänissä
2. Oulun läänissä
3. Itä-Suomen läänissä
4. Länsi-Suomen läänissä
5. Etelä-Suomen läänissä
6. Ahvenanmaalla

**25. Mikä on tämänhetkinen työtilanteesi?**

1. Olen työssä
2. Opiskelen päätoimisesti
3. Olen kotiäiti tai koti-isä
4. Olen tilapäisesti poissa työelämästä (esim. äitiyslomalla, hoito- tai vuorotteluvapaalla)
5. Olen sairauslomalla tai sairauspäivärahalla
6. Olen lomautettuna tai työttömänä
7. Olen eläkkeellä tai osa-aikaeläkkeellä

**27. Onko sinulla itselläsi tällä hetkellä käytössä mitään lääkärin määräämää lääkettä?**

1. Ei (siirry kysymykseen numero 28)
2. Kyllä. Kirjaa kaikki käytössäsi olevat lääkkeet ja niiden käyttötarkoitus.

| Lääkkeen nimi  (esim. Femoden) |  | Käyttötarkoitus  (esim. raskauden ehkäisy) |
| --- | --- | --- |
|  |  |  |
|  |  |  |
|  |  |  |
|  |  |  |
|  |  |  |
|  |  |  |
|  |  |  |
|  |  |  |
|  |  |  |

**18. Mikä on oma äidinkielesi?**

1. Suomi
2. Ruotsi
3. Muu. Mikä? ___________________________________

**20. Syntymävuotesi** _______________

**22. Onko sinulla jokin terveydenhuoltoalan koulutus?**

1. Ei
2. Kyllä. Mikä? ___________________________________

**24. Mikä on koulutuksesi?** Merkitse **ylin** suorittamasi koulutus tai tutkinto.

1. Kansakoulu, osa peruskoulua tai keskikoulua
2. Peruskoulu tai keskikoulu
3. Ammattikoulu tai vastaava
4. Lukio
5. Opistotutkinto
6. Ammattikorkeakoulututkinto
7. Yliopistotutkinto

**26. Mitkä ovat taloutenne yhteenlasketut nettotulot (tulot verojen jälkeen) kuukaudessa?** Jos et tiedä tarkkaa lukua, voit merkitä arviosi.

1. Alle 500 e 7 3000–4999 e
2. 500–999 e 8 5000–7499 e
3. 1000–1499 e 9 7500−10000 e
4. 1500–1999 e 10 Yli 10000 e
5. 2000–2499 e
6. 2500–2999 e

**28. Oletko itse käyttänyt eilen tai toissapäivänä mitään apteekista ilman reseptiä saatavaa lääkettä, mukaan lukien vitamiinit?**

1. En (siirry kysymykseen numero 29)
2. Kyllä. Kirjaa kaikki käyttämäsi lääkkeet ja/tai vitamiinit ja niiden käyttötarkoitus.

| Lääkkeen nimi  (esim. Burana) |  | Käyttötarkoitus  (esim. päänsärky) |
| --- | --- | --- |
|  |  |  |
|  |  |  |
|  |  |  |
|  |  |  |
|  |  |  |
|  |  |  |
|  |  |  |
|  |  |  |
|  |  |  |

**29. Oletko itse käyttänyt eilen tai toissapäivänä ilman reseptiä saatavaa**

**luontaistuotetta, rohdosvalmistetta ja/tai homeopaattista valmistetta?**

1. En (siirry kysymykseen numero 30)
2. Kyllä. Kirjaa kaikki käyttämäsi valmisteet ja niiden käyttötarkoitus.

| Valmisteen nimi |  | Käyttötarkoitus |
| --- | --- | --- |
|  |  |  |
|  |  |  |
|  |  |  |

**30. Seuraavassa on joukko lääkkeitä koskevia väittämiä. Ympyröi mielipidettäsi lähinnä oleva vaihtoehto.**

| **Väittämä** | **Täysin samaa mieltä** | **Jokseenkin samaa**  **mieltä** | **En samaa enkä eri mieltä** | **Jokseenkin eri**  **mieltä** | **Täysin**  **eri**  **mieltä** | **En**  **osaa**  **sanoa** |
| --- | --- | --- | --- | --- | --- | --- |
|  |  |  |  |  |  |  |
| Lääkkeet ovat välttämättömiä sairauksien hoidossa. | 5 | 4 | 3 | 2 | 1 | 0 |
| Lapselle annettavien lääkkeiden haittavaikutukset huolestuttavat minua. | 5 | 4 | 3 | 2 | 1 | 0 |
| Pyrin välttämään lääkkeiden käyttöä lapselle. | 5 | 4 | 3 | 2 | 1 | 0 |
| Ilman reseptiä saatavat lääkkeet, eli itsehoitolääkkeet, ovat turvallisia. | 5 | 4 | 3 | 2 | 1 | 0 |
| Lapsen elimistön luonnollista puolustuskeinoa, kuumetta, ei pidä alentaa lääkkeillä keinotekoisesti. | 5 | 4 | 3 | 2 | 1 | 0 |
| Lapsen pitää oppia kestämään kipua. | 5 | 4 | 3 | 2 | 1 | 0 |
| Reseptilääkkeet ovat tehokkaita. | 5 | 4 | 3 | 2 | 1 | 0 |
| Annan lapselle yleensä särkylääkettä vähemmän kuin ohjeessa suositellaan. | 5 | 4 | 3 | 2 | 1 | 0 |
| Reseptilääkkeet ovat turvallisia. | 5 | 4 | 3 | 2 | 1 | 0 |
| Lääkkeet saattavat häiritä elimistön omaa paranemiskykyä. | 5 | 4 | 3 | 2 | 1 | 0 |
| Lääkärin lapselle määräämät lääkkeet ovat tarpeellisia. | 5 | 4 | 3 | 2 | 1 | 0 |
| Lääkkeet ovat epäluonnollisia ihmisen elimistölle. | 5 | 4 | 3 | 2 | 1 | 0 |
| Pyrin hoitamaan lapsen vaivan muuten kuin lääkkeillä. | 5 | 4 | 3 | 2 | 1 | 0 |
| Mitä enemmän särkylääkkeitä joutuu käyttämään, sitä huonommin ne tehoavat kipuun. | 5 | 4 | 3 | 2 | 1 | 0 |
| Hoidan lapsen pienet vaivat itsehoitolääkkeillä. | 5 | 4 | 3 | 2 | 1 | 0 |
| Lääkkeet ovat ohjeen mukaan käytettynäkin vaarallisia. | 5 | 4 | 3 | 2 | 1 | 0 |
| Vien lapsen lääkäriin vasta, kun muut hoitokeinot eivät riitä. | 5 | 4 | 3 | 2 | 1 | 0 |
| Särkylääkkeiden pitkäaikainen käyttö alentaa kipukynnystä. | 5 | 4 | 3 | 2 | 1 | 0 |
| Itsehoitolääkkeet ovat tehokkaita. | 5 | 4 | 3 | 2 | 1 | 0 |
| Lääkärit määräävät antibiootteja lapsille liian helposti. | 5 | 4 | 3 | 2 | 1 | 0 |
| Lääkkeiden yhteisvaikutukset huolestuttavat minua. | 5 | 4 | 3 | 2 | 1 | 0 |

Voit halutessasi kirjoittaa tähän ajatuksiasi lapsen lääkehoidosta. Voit myös kirjoittaa lapsen lääkehoitoon liittyvistä ongelmista ja huolista. Niistä saamme arvokasta tietoa tutkimukseemme.

**____________________________________________________________________________________________________________________________________________________________________________________________________________________________________________________________________________________________________________________________________________________________**

**____________________________________________________________________________________________________________________________________________________________________________________________________________________________________________________________________________________________________________________________________________________________**

**____________________________________________________________________________________________________________________________________________________________________________________________________________________________________________________________________________________________________________________________________________________________**

**____________________________________________________________________________________________________________________________________________________________________________________________________________________________________________________________________________________________________________________________________________________________**

**____________________________________________________________________________________________________________________________________________________________________________________________________________________________________________________________________________________________________________________________________________________________**

**____________________________________________________________________________________________________________________________________________________________________________________________________________________________________________________________________________________________________________________________________________________________**

**____________________________________________________________________________________________________________________________________________________________________________________________________________________________________________________________________________________________________________________________________________________________**

**____________________________________________________________________________________________________________________________________________________________________________________________________________________________________________________________________________________________________________________________________________________________**

**Kiitos!**
