# Supplementary material for: Children's health and parental socioeconomic factors: a population-based survey in Finland
Source: BMC Public Health. 2011 Jun 9;11:457. doi: 10.1186/1471-2458-11-457 (PMC3135536; doi:10.1186/1471-2458-11-457)
Supplement: Additional file 2 — Survey questionnaire Original questionnaire used in the study, translated into English [file 1471-2458-11-457-S2.DOC]

The Faculty of Pharmacy

University of Kuopio 2007

**CHILDREN’S MEDICINE USE**

**Answer the questions by circling the best fitting alternative or writing down in the blank space. Your answers are important even if the child is not using any medicines.**

**BACKGROUND INFORMATION OF THE CHILD**

**1. The child is a**

1. Girl
2. Boy

**2. Date of birth**

day month year

**3. Is the child**

1. The firstborn
2. The second born
3. The third born
4. Other.____________

**4. Which answer best describes the child’s usual care during the day? (choose the one which they spend the most hours at per week)**

1. Goes to school or preschool
2. Is taken care of by a child minder outside home
3. Is in the day-care-centre
4. Is taken care of at home alone or with brothers and sisters
5. Some other way. How?

_______________________________________________

_______________________________________________

**HEALTH STATUS OF THE CHILD**

**5. What is your opinion about the health status of the child at the moment?**

1. Good
2. Fairly good
3. Moderate
4. Fairly poor
5. Poor

**6. Does the child have any illness or injury that has been diagnosed by a doctor?**

1. No
2. Yes, what illness/illnesses?

_____________________________________________ _____________________________________________

**7. Below is a list of symptoms. Circle for each symptom if the child is suffering it at the moment.**

| **Symptoms** | **No** | **Yes** | **Don’t know** |
| --- | --- | --- | --- |
|  |  |  |  |
| Constipation | 1 | 2 | 3 |
| Diarrhoea | 1 | 2 | 3 |
| Stomach bug | 1 | 2 | 3 |
| Flatulence/Wind | 1 | 2 | 3 |
| Other stomach disorders, describe?____________________ | 1 | 2 | 3 |
| Headache | 1 | 2 | 3 |
| Pain in the neck or shoulders | 1 | 2 | 3 |
| Pain in the lower back | 1 | 2 | 3 |
| Earache | 1 | 2 | 3 |
| Sore throat | 1 | 2 | 3 |
| Growing pains | 1 | 2 | 3 |
| Other pain, describe? __________________________ | 1 | 2 | 3 |
| High temperature/Fever | 1 | 2 | 3 |
| Symptoms of cold/flu (e.g. runny nose, cough) | 1 | 2 | 3 |
| Allergic symptoms (e.g. runny nose, eye symptoms) | 1 | 2 | 3 |
| Eczema or skin symptoms | 1 | 2 | 3 |
| Fatigue or feeling faint | 1 | 2 | 3 |
| Sleep disturbance | 1 | 2 | 3 |
| Tension or nervousness | 1 | 2 | 3 |
| Low spirit or depression | 1 | 2 | 3 |
| Some other symptom, describe? ______________________ | 1 | 2 | 3 |
|  |  |  |  |

**THE CHILD’S MEDICINE USE**

**8. Is the child using any prescription medicines at the moment?**

1. No (move to the question number 9)
2. Yes. List every medicine and its purpose of use. List also those medicines that are used as needed. You can continue on the last page.

| Name of the medicine  (e.g. Flixotide® Inhaler) |  | Purpose of use  (e.g. asthma) |
| --- | --- | --- |
|  |  |  |
|  |  |  |
|  |  |  |
|  |  |  |
|  |  |  |

**9. Has your child taken yesterday or the day before yesterday any Over the counter (OTC) medicines, including vitamins?**

1. No (move to the question number 10)
2. Yes. List every medicine and its purpose of use.

| Name of the medicine  (e.g. Sigmacort® 1% cream) |  | Purpose of use  (e.g. eczema) |
| --- | --- | --- |
|  |  |  |
|  |  |  |
|  |  |  |
|  |  |  |
|  |  |  |

**10. Is the child using any OTC medicines or vitamins daily or almost daily?**

1. No (move to the question number 11)
2. Yes. List all the medicines that are used daily or almost daily, and also how long the child has been using them.

| Name of the product |  | How long has (s)he been  using it? /Length of use |
| --- | --- | --- |
|  |  |  |
|  |  |  |
|  |  |  |
|  |  |  |
|  |  |  |

**11. Has the child taken yesterday or the day before yesterday any medicinal herbs, botanicals, or homeopathic products?**

1. No (move to the question number 12)
2. Yes. List all products and their purpose of use.

| Product |  | Purpose of use |
| --- | --- | --- |
|  |  |  |
|  |  |  |
|  |  |  |
|  |  |  |
|  |  |  |

**12. Has *any* medicine caused the child harm?**

1. No (move to the question number 13)
2. Yes. List all the harms and medicines that have caused them. You can continue on the last page.

_________________________________________________________________________________________________________________________________________________________________________________________________________________________________________________________________________

**13. Have there been any other problems with the child’s medication?**

1. No (move to the question number 14)
2. Yes. What kind of problems? List also which medicines have caused them. You can continue on the last page.

_________________________________________________________________________________________________________________________________________________________________________________________________________________________________________________________________________

**14. At what age, on your opinion, can the child decide on taking a medicine for little ailments, e.g., headache, independently without any control or guidance of a parent?**

1. < 4
2. 4–5
3. 6–7
4. 8–9
5. 10–11
6. 12–13
7. 14–15
8. 16–17
9. 18 or older
10. No opinion

**INFORMATION SOURCES OF MEDICINES**

**15. Below is a list of information sources. Circle for every source how much you have used it when searching information concerning children's medication.**

| **Source of information** | **Much** | **To some extent** | **Little** | **Not at all** |
| --- | --- | --- | --- | --- |
|  |  |  |  |  |
| Doctor/Physician | 3 | 2 | 1 | 0 |
| Nurse | 3 | 2 | 1 | 0 |
| Public health nurse / School health nurse | 3 | 2 | 1 | 0 |
| Relative or friend who is a health care professional | 3 | 2 | 1 | 0 |
| Pharmacist | 3 | 2 | 1 | 0 |
| Helpline, which one?___________________________ | 3 | 2 | 1 | 0 |
| Patient information leaflet | 3 | 2 | 1 | 0 |
| Brochures concerning medicines | 3 | 2 | 1 | 0 |
| Medical books | 3 | 2 | 1 | 0 |
| Some other book, which one? ____________________ | 3 | 2 | 1 | 0 |
| Health journals | 3 | 2 | 1 | 0 |
| Ordinary newspapers or magazines | 3 | 2 | 1 | 0 |
| Radio, television | 3 | 2 | 1 | 0 |
| The internet, which websites?_____________________ | 3 | 2 | 1 | 0 |
| Family, friends | 3 | 2 | 1 | 0 |
| Health food shop /Natural product store | 3 | 2 | 1 | 0 |
| Other, what?_________________________________ | 3 | 2 | 1 | 0 |
|  |  |  |  |  |

**16. How reliable do you find the following information sources when searching information concerning children's medication?**

| **Source of information** | **Very reliable** | **Reliable** | **Quite reliable** | **Not**  **reliable** | **No**  **opinion or haven’t used** |
| --- | --- | --- | --- | --- | --- |
|  |  |  |  |  |  |
| Doctor/Physician | 4 | 3 | 2 | 1 | 0 |
| Nurse | 4 | 3 | 2 | 1 | 0 |
| Public health nurse / School health nurse | 4 | 3 | 2 | 1 | 0 |
| Relative or friend who is a health care professional | 4 | 3 | 2 | 1 | 0 |
| Pharmacist | 4 | 3 | 2 | 1 | 0 |
| Helpline, which one?___________________________ | 4 | 3 | 2 | 1 | 0 |
| Patient information leaflet | 4 | 3 | 2 | 1 | 0 |
| Brochures concerning medicines | 4 | 3 | 2 | 1 | 0 |
| Medical books | 4 | 3 | 2 | 1 | 0 |
| Some other book, which one? ____________________ | 4 | 3 | 2 | 1 | 0 |
| Health journals | 4 | 3 | 2 | 1 | 0 |
| Ordinary newspapers or magazines | 4 | 3 | 2 | 1 | 0 |
| Radio, television | 4 | 3 | 2 | 1 | 0 |
| The internet, which website?_____________________ | 4 | 3 | 2 | 1 | 0 |
| Family, friends | 4 | 3 | 2 | 1 | 0 |
| Health food shop /Natural product store | 4 | 3 | 2 | 1 | 0 |
| Other, what?_________________________________ | 4 | 3 | 2 | 1 | 0 |
|  |  |  |  |  |  |

**BACKGROUND INFORMATION**

**17. Who is the person that filled in this questionnaire?**

1. Mother
2. Father
3. Other. Who?_________________________

**18. What is your native language?**

1. Finnish
2. Swedish
3. Other - please list________________________________

**19. Number of children in the family** ___________________

**20. Your year of birth** ___________

**21. Did the child take part in filling in this questionnaire?**

1. No
2. Yes

**22. Have you taken any degree in health care?**

1. No
2. Yes. Which one? ________________________________ ______________________________________________

**23. Which province do you live in?**

1. Lapland
2. Oulu
3. Eastern Finland
4. Western Finland
5. Southern Finland
6. Åland

**24. What is your level of education? Circle the highest degree you have taken.**

1. Primary school
2. School certificate (equivalent to year ten)
3. Higher School Certificate (leaving certificate/year 12)
4. Non University Diploma
5. Bachelors Degree (University)
6. Postgraduate University Degree

**25. What is your current employment status? (Choose the one best option that describes your situation)**

1. I’m working part-time/full time
2. I study or go to school
3. I’m a housewife/-husband
4. I’m temporarily absent from work (for example on maternity leave)
5. I’m on sick leave
6. I have been laid off or I’m unemployed
7. I’m retired

**26. How much is your household’s net income/month?**

1. Below 500 e
2. 500–999 e
3. 1000–1499 e
4. 1500–1999 e
5. 2000–2499 e
6. 2500–2999 e
7. 3000–3999 e
8. 4000–4999 e
9. 5000–7499 e
10. 7500–10000 e
11. Over 10 000 e

**27. Are you yourself using any prescription medicines at the moment?**

1. No (move to the question no 28)
2. Yes. List all the medicines that you are using and their purpose of use.

| Medicine  (e.g. Trifeme®) |  | Purpose of use  (e.g. contraception) |
| --- | --- | --- |
|  |  |  |
|  |  |  |
|  |  |  |
|  |  |  |
|  |  |  |
|  |  |  |

**28. Have you taken yesterday or the day before yesterday any Over the counter (OTC) medicines, including vitamins?**

1. No (move to the question no 29)
2. Yes. List the name of the medicine and what you used it for.

| Medicine  (e.g. Nurofen®) |  | Purpose of use  (e.g. headache) |
| --- | --- | --- |
|  |  |  |
|  |  |  |
|  |  |  |
|  |  |  |
|  |  |  |
|  |  |  |

**29. Have you taken yesterday or the day before yesterday any medicinal herbs, botanicals, or homeopathics?**

1. No (move to the question no 30)
2. Yes. List the name of the product and what you used it for.

| Product |  | Purpose of use |
| --- | --- | --- |
|  |  |  |
|  |  |  |
|  |  |  |
|  |  |  |

**30. Below is a list of statements. Circle the option nearest to your own opinion.**

| **Statement** | **I agree completely** | **I agree** | **I don’t agree or disagree** | **I disagree** | **I disagree completely** | **No opinion** |
| --- | --- | --- | --- | --- | --- | --- |
|  |  |  |  |  |  |  |
| Medicines are necessary in treating illnesses. | 5 | 4 | 3 | 2 | 1 | 0 |
|  |  |  |  |  |  |  |
| Side-effects of children’s medicines worry me. | 5 | 4 | 3 | 2 | 1 | 0 |
|  |  |  |  |  |  |  |
| I try to avoid giving medicines to my child. | 5 | 4 | 3 | 2 | 1 | 0 |
|  |  |  |  |  |  |  |
| Over the counter (OTC) medicines are safe. | 5 | 4 | 3 | 2 | 1 | 0 |
|  |  |  |  |  |  |  |
| Fever, a natural means of defense of the child’s body, should not be lowered artificially with medicines. | 5 | 4 | 3 | 2 | 1 | 0 |
|  |  |  |  |  |  |  |
| The child needs to learn how to bear the pain. | 5 | 4 | 3 | 2 | 1 | 0 |
|  |  |  |  |  |  |  |
| Prescription medicines are effective. | 5 | 4 | 3 | 2 | 1 | 0 |
|  |  |  |  |  |  |  |
| I usually give less analgesic to the child than is recommended in the instructions. | 5 | 4 | 3 | 2 | 1 | 0 |
|  |  |  |  |  |  |  |
| Prescription medicines are safe. | 5 | 4 | 3 | 2 | 1 | 0 |
|  |  |  |  |  |  |  |
| Medicines can disturb the body’s own capability to heal illnesses. | 5 | 4 | 3 | 2 | 1 | 0 |
|  |  |  |  |  |  |  |
| Medicines that a doctor has prescribed for the child are necessary. | 5 | 4 | 3 | 2 | 1 | 0 |
|  |  |  |  |  |  |  |
| Medicines are unnatural to the human body. | 5 | 4 | 3 | 2 | 1 | 0 |
|  |  |  |  |  |  |  |
| I try to take care of my child’s ailments by some other means than using medicines. | 5 | 4 | 3 | 2 | 1 | 0 |
|  |  |  |  |  |  |  |
| The more you need to use analgesics the less effective they are for pain. | 5 | 4 | 3 | 2 | 1 | 0 |
|  |  |  |  |  |  |  |
| I take care of my child’s minor ailments by using OTC medicines. | 5 | 4 | 3 | 2 | 1 | 0 |
|  |  |  |  |  |  |  |
| Medicines are dangerous, even when used according to the instructions. | 5 | 4 | 3 | 2 | 1 | 0 |
|  |  |  |  |  |  |  |
| I take my child to see a doctor only when other ways of treatment do not help. | 5 | 4 | 3 | 2 | 1 | 0 |
|  |  |  |  |  |  |  |
| Long-term use of analgesics reduces the pain threshold. | 5 | 4 | 3 | 2 | 1 | 0 |
|  |  |  |  |  |  |  |
| OTC medicines are effective. | 5 | 4 | 3 | 2 | 1 | 0 |
|  |  |  |  |  |  |  |
| Doctors prescribe antibiotics to children too easily. | 5 | 4 | 3 | 2 | 1 | 0 |
|  |  |  |  |  |  |  |
| Interactions of medicines worry me | 5 | 4 | 3 | 2 | 1 | 0 |

**THANK YOU!**

**Last page for free comments.**

________________________________________________________________________________________________________________________________________________________________________________________________________________________________________________________________________________________________________________________________________________________________________________________________________________________________________________________________________________________________________________________________________________________________________________________________________________________________________________________________________________________________________________________________________________________________________________________________________________________________________________________________________________________________________________________________________________________________________________________________________________________________________________________________________________________________________________________________________________________________________________________________________________________________________________________________________________________________________________________________________________________________________________________________________________________________

________________________________________________________________________________________________________________________________________________________________________________________________________________________________________________________________________________________________________________________________________________________________________________________________________________________________________________________________________________________________________________________________________________________________________________________________________________________________________________________________________________________________________________________________________________________________________________________________________________________________________________________________________________________________________________________________________________________________________________________________________________________________________________________________________________________________________________________________________________________________________________________________________________________________________________________________________________________________________________________________________________________________________________________________________________________________

__________________________________________________________________________________________________________________________________________________________________________________________________________________________________________________________________
